# *Bacillus amyloliquefaciens* CU33 Fermented Product Improves Growth Performance, Diarrhea, and Immunity of Goat Kids

**DOI:** 10.3390/ani15091324

**Published:** 2025-05-03

**Authors:** Tsung-Yu Lee, Yueh-Sheng Lee, Chean-Ping Wu, Bor-Chun Weng, Kuo-Lung Chen

**Affiliations:** 1 Animal Nutrition Division, Taiwan Livestock Research Institute, Ministry of Agriculture, Tainan 712009, Taiwan; johntyli@mail.tlri.gov.tw; 2 Chiaho Agrscience Co., Ltd., Chiayi 600039, Taiwan; lopqkojhog@gmail.com; 3 Department of Animal Science, National Chiayi University, Chiayi 600355, Taiwan; wcp@mail.ncyu.edu.tw; 4 Department of Microbiology, Immunology and Biopharmaceuticals, National Chiayi University, Chiayi 600355, Taiwan; brian@mail.ncyu.edu.tw

**Keywords:** *Bacillus amyloliquefaciens* CU33, fermentation, goat kids, growth performance, immunity

## Abstract

This study focused on the physicochemical characteristics of the fermented products (FP) produced by *Bacillus amyloliquefaciens* CU33 (CU33) from soybean meal with 70% moisture. Additionally, it investigated the effects of adding FP to starter on the growth performance, general health performance, blood clinical biochemistry, and immunity of Alpine goat kids during the weaning period. Forty 14-day-old male Alpine goat kids were randomly assigned into starter supplementations of 0, 0.1, 0.3, or 0.5% CU33 FP for 8 weeks, and each goat kid was individually raised in stainless steel cage (width 70 cm × height 70 cm × depth 80 cm). The moisture after fermentation was linearly decreased as fermentation time increased (*p* < 0.05), and the pH value and *Bacillus*-like counts reached the highest at 24 h of fermentation. The activity of neutral protease and alkaline protease, the content of surfactin and γ-PGA, the viscosity, and the odor of CU33 FP were linearly increased as fermentation time increased (*p* < 0.05). The neutral protease activity, surfactin, γ-PGA, and viscosity increased after drying, whereas the moisture, pH value, *Bacillus*-like counts, and odor decreased (*p* < 0.05). During the pre-weaning period (0–4 weeks), the body weight gain (BWG) of the 0.1% CU33 FP group was higher than that of the control group (*p* < 0.05), and all CU33 FP groups showed a better feed conversion ratio (FCR) than the control group (*p* < 0.05). During the post-weaning period (4–8 weeks) and throughout the entire experimental period (0–8 weeks), the BWG and FCR of all CU33 FP groups were better than those of the control group (*p* < 0.05). Furthermore, both BWG and FCR improved linearly as the dietary level of CU33 FP increased (*p* < 0.05). Simultaneously, the fecal consistency index at 0–4 and 4–8 weeks and the coliform counts in the rectum at 4 weeks linearly decreased (*p* < 0.05), and the *Bacillus*-like counts in the rectum linearly increased at 4 and 8 weeks (*p* < 0.05). Phosphorous (P), total protein (TP), blood urea nitrogen (BUN) in serum at 8 weeks, and the oxidative burst capacity at 4 weeks linearly increased as the dietary level of CU33 FP increased, but the skin sensitization test showed a quadratic curve, and the 0.1% CU33 FP group had the lowest performance (*p* < 0.05). In conclusion, dietary supplementation with 0.1% of CU33 FP can improve the growth performance, diarrhea status, and oxidative burst capacity of Alpine goat kids, showing the potential to be a feed additive.

## Simple Summary

This study investigated a fermented product (FP) made from soybean meal using *Bacillus amyloliquefaciens* CU33 (CU33) and its effects on the growth and health of Alpine goat kids during weaning. Forty 14-day-old male Alpine goat kids were fed diets supplemented with 0, 0.1%, 0.3%, or 0.5% CU33 FP for eight weeks. Fermentation reduced moisture while increasing beneficial compounds like protease enzymes, surfactin, and γ-PGA. Adding CU33 FP to the diet improved body weight gain and feed efficiency, with better results as the inclusion level increased. The 0.1% CU33 FP group showed the highest weight gain before weaning, and all FP groups had better feed conversion ratios than the control. Additionally, CU33 FP enhanced gut health by increasing beneficial *Bacillus* counts and reducing harmful coliform bacteria. It also improved blood phosphorus, total protein, and immune response, lowering diarrhea incidence. These findings suggest that CU33 FP can be a valuable dietary supplement for improving weaning Alpine goat kids’ growth, digestion, and immunity.

## 1. Introduction

Early weaning to reduce the cost of raising meat goats is a common method in Taiwan. The ram kids of dairy goats are sold 2–3 after days of being born for meat and fed artificial milk until 6–12 weeks of age. Therefore, it is easy to have poor growth and diarrhea symptoms under transportation, changes in diet, and stressful conditions, also combined with complex infections and death, resulting in economic losses for the industry. There are different opinions on the feeding and management conditions around the weaning of goat kids. Generally, the weight (e.g., 14–15 kg, or 2–2.5 times the birth weight), age (at 6–8 weeks of age), or feed intake (e.g., starter intake 115–200 g/d, or feed intake including hay 30–500 g/d) are as a basis [1]. Furthermore, the goat kids transition from a liquid to a solid diet during this period [2], and this will affect the reduction of endocrine and metabolism functions to slow down weight gain [3,4].

Goat kids consume starter has positive effect on rumen growth [5], and they can usually be provided ad libitum at 1–3 weeks of age to serve as a bridge of feed around weaning [1,6,7]. Probiotics can improve intestinal microbial balance, intestinal digestion, and animal growth, and they are widely used in the food and feed industries [8,9,10]. Adding *Bacillus subtilis* to the diet or milk replacer benefits the growth and immunity of calves and lambs [11,12,13]. Supplementation of *Bacillus amyloliquefaciens*-9 in the milk replacer can maintain intestinal microbial homeostasis of Saanen goat kids to improve gut health [14]. The use of solid-state fermentation technology can enhance the level of probiotics, enzymes, and beneficial secondary metabolites, and also can convert certain compounds into more effective ingredients [15,16,17]. Hong and Wu found that adding two-stage fermented feather meal with various *Bacillus* spp. to the diet can improve the growth performance of goat kids [7]. The authors screened out *Bacillus amyloliquefaciens* CU33 (CU33) with strong feather decomposition ability. Subsequently, it was aerobically fermented with feather meal–soybean meal for 2 days. Supplementation with the fermented product in the broiler diet could significantly improve the villus morphology of duodenum and the feed conversion ratio [10]. Adding 1% of the product could improve protein digestion, diarrhea, immunity, and the growth performance of goat kids [18].

CU33 has the ability to secrete poly-γ-glutamic acid (γ-PGA), enzymes (e.g., protease, amylase, and lipase), and surfactin; thus, it has the potential to be an animal feed additive. Surfactin can also inhibit *Escherichia coli*, which has antibacterial and anti-biofilm properties [19]. If the contents of the above products are increased during fermentation, it will improve the production and economic benefits. Therefore, changing the fermented substrates with full soybean meal (not feather meal–soybean meal) increased the moisture to 70%. This study investigated the physical and chemical properties of CU33 fermented product and the effect of it as a starter on the growth, health, blood, and immune characteristics of goat kids around weaning.

## 2. Materials and Methods

### 2.1. Fermented Product Preparation

The fermentation process refers to the method of Lee et al. [10]; the moisture of the soybean meal was adjusted to 65% and sterilized at 121 °C, 1.21 kg/cm^2^ for 20 min. Then 5% *B. amyloliquefaciens* CU33 (10^7^ CFU/g feed) was after it cooled down, and aerobic fermentation was conducted at 37 °C for 48 h. The fermented product was dried in an oven at 55 °C, which was CU33 fermented product (CU33FP).

### 2.2. The Physiochemical Characterizations and Nutrient Composition of CU33FP

The pH of CU33FP was measured by a portable pH meter (digital pH meter, Goodly, Taiwan). The viscosity of CU33FP was evaluated on a 5-point scale where 1 is the best score (viscosity: 1 = not sticky; odor: 1 = most acceptable) and 5 is the worst. The CU33FP was serially diluted in 0.85% NaCl and incubated on tryptic soy agar (TSA, HIMEDIA^®^, Mumbai, MH, India) at 37 °C for 24 h or on potato dextrose agar (PDA, HIMEDIA^®^, Mumbai, MH, India) at 28 °C for 48 h, respectively, in colony counting for *Bacillus*-like colonies. The γ-PGA of CU33FP was measured by the method of Goto and Kunioka [20]. The approximate analysis of CU33FP followed the description of AOAC [21] to analyze the moisture (method 930.15), crude protein (CP) (method 990.03), crude ash (method 942.05), calcium (Ca) (method 927.02), and phosphorus (P) (935.59) of feed. The gross energy was measured with an adiabatic bomb calorimeter (model 356, Parr Instrument Company, Moline, IL, USA). The protease activity of CU33FP refers to the methods of Secades and Guijarro [22] and Oguntoyinbo et al. [23]. The surfactin yield of CU33FP was measured by the method of Lee et al. [24].

### 2.3. Animal Management and Experimental Design

Forty 14-day-old Alpine goat kids (male) were randomly assigned to dietary supplementation groups of 0, 0.1, 0.3, and 0.5% CU33FP. Each treatment had ten replicates. The experimental period was 8 weeks. After the goat kids were fed with colostrum for 3 days, they were fed with the milk replacer (Victoria Whole Milk Powder, Victoria, Australia) (CP 24.5%, fat 26.3%, lactose 40.3%, mineral 5.8%, and moisture 3.1%) until 6 weeks of age. The milk replacer was reduced to 136 g DM/L milk replacer at 38 °C for the entire experiment. The feeding method of milk replacer was 3 times a day in the first week of the trial period with 900 mL, 3 times a day in the second week of the trial period with 600 mL, twice a day in the third week of the trial period with 400 mL, and one time a day in the fourth week of the trial period with 200 mL, then completely weaned at the end of the fourth week of the experiment (at 6 weeks old). The starter refers to Lee et al. [18]; the formula is shown in Table 1. The starter was fed to them twice a day, and the water was taken freely. The kids were individually raised in stainless steel cages (width 70 cm × height 70 cm × depth 80 cm). The experiment was approved by the ethics Committee of the Institutional Animal Care and Use Committee of National Chiayi University (111046).

### 2.4. Growth Performance

The feed intake (FI) of milk replacer and starter were recorded daily, the body weights (BW) of goat kids were weighed weekly, and weight gain (WG) and feed conversion ratio (FCR) were calculated throughout the experiment.

### 2.5. General Health Performance

During the experiment period, we recorded the number of diarrhea episodes, the duration of diarrhea, the diarrhea rate, and the number of therapeutic treatments administered to the goat kids on a daily basis. The incidence of various diseases was assessed based on the number of antibiotic treatments prescribed by the veterinarian for digestive, respiratory, or other conditions such as joint or umbilical infections. The General Health Score (GHS) was measured according to the method described by Timmerman et al. [25], with some modifications. The incidence of diarrhea and the therapeutic treatments for digestive, respiratory, and other diseases was weighted differently by the following formula: GHS per animal = 28 − (1 × total number of days of diarrhea, regardless of type) − (2 × number of individual therapeutic treatments for digestive diseases) − (3 × number of individual therapeutic treatments for respiratory diseases) − (2 × number of individual therapeutic treatments for infections other than digestive or respiratory) − (2 × number of antibiotic treatments on a herd basis). The weighting factors for each condition were determined based on their assumed impact on health.

The fecal appearance and consistency were recorded, scored, and assigned four values according to the fecal consistency index (FCI) modified by Meyer et al. [26]: (1) Normal—(dE1): Feces are solid but not hard, slightly deformed when dropped and placed on the floor. (2) Soft—(dE2): feces unformed, piled up, and scattered when falling. (3) Liquid—(dE3): feces dispersed in 6 mm deep flakes. (4) Water—(dE3): feces with liquid consistency.

(1) $$\mathrm{FCI}=\frac{\left(\mathrm{dE}1\times 1\right)+\left(\mathrm{dE}2\times 2\right)+\left(\mathrm{dE}3\times 3\right)+\left(\mathrm{dE}4\times 4\right)}{\mathrm{Td}\;\times 4}\times 100$$

where dE1, dE2, dE3, and dE4 are the days with fecal consistency index = 1, 2, 3, and 4, respectively, and Td is the number of the trial period days (Td = 28).

The Count of Bacteria in Feces: At weeks 4 and 8 of the experiment, fecal samples were collected from six goat kids per treatment group. Prior to collecting the samples, the anal area of each goat kid was cleaned three times using wipes and 75% alcohol. Approximately 1 g of fecal material was then collected using sterilized plastic gloves and placed in a 50 mL sterilized centrifuge tube for refrigeration in an ice bath. The sample was subsequently diluted tenfold with buffered peptone water, and the contents were shaken for 2 min. The supernatant was then obtained and subjected to serial dilution. A volume of 0.1 mL was applied to Violet Red Bile Lactose Agar (VRBLA, HIMEDIA^®^, Mumbai, MH, India) and Tryptone Soya Agar (TSA, HIMEDIA^®^, Mumbai, MH, India). The plates were incubated at 37 °C for 24 h to determine the colony numbers (CFU/g feces) of coliforms and *Bacillus*-like bacteria.

### 2.6. Hematological Traits

At the end of the experiment, blood samples were collected from the jugular vein using an EDTA vacutainer. A total of 8 mL of blood was obtained from 10 goat kids per treatment and stored at 4 °C until analysis. Various blood profiles were assessed, including white blood cells (WBCs), red blood cells (RBCs), hemoglobin (Hgb), hematocrit (Hct), mean corpuscular volume (MCV), mean corpuscular hemoglobin (MCH), mean corpuscular hemoglobin concentration (MCHC), platelets (PLTs), leukocytes, neutrophil extracellular traps (Net-s), lymphocytes (Lym-L), monocytes (Mono), eosinophils (Eos), and basophils (Baso). These parameters were measured using an automatic blood analyzer (ADVIA 120, Bayer, NY, USA). Serum haptoglobin levels were determined using an enzyme-linked immunosorbent assay kit (plate number 1; Tri-Delta Diagnostics, Morris Plains, NJ, USA). For hematology analysis, approximately 3 mL of blood was collected in tubes containing sodium heparin (BD Medical, Vacutainer number 1, Becton Dickinson, Franklin Lakes, NJ, USA) and analyzed immediately after collection. Blood samples for haptoglobin determination were placed in serum separator tubes (BD Vacutainer, Becton Dickinson, Franklin Lakes, NJ, USA), centrifuged at 1500× *g* for 10 min, and then stored at −80 °C until assayed.

### 2.7. Clinical Blood Biochemistry

At the end of the experiment, blood samples were collected from the jugular vein of goat kids. The samples were centrifuged at 1700× *g* for 15 min, and the serum was stored at −40 °C for subsequent analysis. The biochemistry of the serum was examined, focusing on the activities of various enzymes including aspartate aminotransferase (AST), alanine aminotransferase (ALT), γ-glutamyl transpeptidase (γ-GT), lactate dehydrogenase (LDH), creatine kinase (CK), alkaline phosphatase (ALP), and acid phosphatase (Acid-P). Additionally, the concentrations of several substances were measured: calcium (Ca), phosphate (P), glucose, cholesterol, triglycerides (TGs), total protein (TP), albumin (Alb), globulin (Glo), the albumin/globulin ratio (A/G), blood urea nitrogen (BUN), and creatinine. All analyses were conducted using an automatic blood chemical analyzer with Roche testing kits (Roche COBAS MIRA PLUS, Basel, Switzerland).

### 2.8. Isolation of Peripheral Blood Mononuclear Cells and Granulocytes

Separation and purification of phagocytes and lymphocytes: The goat blood sample was centrifuged at 1000× *g* for 10 min to reveal three layers (serum in the upper layer, lymphocytes in the middle layer, and granulocyte in the under layer). Serum was stored at −80 °C. After the middle layer of blood was taken out, it was slowly added to 15 mL centrifuge tube containing Ficoll-Paque (density 1.077), then slow-speed centrifuged at 300× *g* for 30 min to purify lymphocytes for assays. The underlayer of blood was subsequently lysed by commercial RBC-lysis buffer (BioLegend, San Diego, CA, USA) to remove red blood cells and purify phagocytes for the following assays.

### 2.9. Phagocytosis of Granulocyte

Phagocytosis of granulocyte: granulocytes of 1 × 10^6^ each were preseeded in a 96-well plate and then cocultured with fluorescently labeled bacteria at 1 × 10^7^ DioC18-labeled *E. coli* (ATCC 25922) in a PBS solution at 37 °C for 3 h. By the end of the coincubation, the fluorescence of FL-1 was determined by flow cytometry (Becton Dickinson FACSCaliburTM, Franklin Lakes, NJ, USA) to collect data, and we used WinMDI 2.8 software for analysis.

### 2.10. Oxidative Burst Measurement

The measurement of the oxidative burst followed the protocol outlined by Ciapetti et al. [27]. Granulocytes were co-incubated with unlabeled *E. coli* at 37 °C for 90 min. To determine the levels of intracellular reactive oxygen species (ROS), 2′,7′-dichlorofluorescin-diacetate (DCF-DA) was added. The amount of DCF-DA produced was directly proportional to the ROS generated during the oxidative burst of the granulocytes, and this process was measured using a flow cytometer.

### 2.11. Lymphoblastogenesis

The lymphocytes of 2 × 10^5^ were inoculated in a 96-well plate for 24 h. Subsequently, specific mitogens were added, respectively, for inoculated 24 h, such as phytohaemagglutinin (PHA) or lipopolysaccharide (LPS), all purchased from Sigma, USA. After that, 20 µL Alamar Blue (Serotec Co., Oxford, UK) was added. The enzyme (NADH) in the mitochondria of the cell would reduce Alamar Blue, the original dark blue and non-fluorescent, to a pink, highly fluorescent product, then we cultured it in the incubator to observe its color change. The excitation light at 528 nm and the scattered light at 590 nm were measured in the reaction results by a fluorescence/luminescence analyzer (Thermo Scientific Varioskan LUX, Level Biotechnology Inc., Hsinchu, Taiwan).

### 2.12. Skin Swelling Measurement

Ten goat kids from each treatment group were selected, injected with 0.05 mL of phytohaemagglutinin (PHA) into the skin of their left ear, and injected with 0.05 mL of normal saline into the skin of the right ear of the goat kids as a control group. After 12 h, we measured the degree of swelling of the wattle by a digital thickness gauge.

### 2.13. Blood Immunoglobulin Level

To determine the concentrations of IgG, IgM, and IgA, a commercial ELISA kit from Bethyl Laboratories (Montgomery, TX, USA) was used at 4 and 8 weeks.

### 2.14. Statistical Analysis

The data were analyzed using the Generalized Linear Model (GLM) procedure [28], and the groups were compared with a one-way ANOVA followed by a Tukey post hoc test. A *p*-value of less than 0.05 was considered to indicate a statistically significant difference. The General Health Scores (GHSs) and Functional Capacity Index (FCI) related to general health performance were evaluated using the NPAR1WAY procedure [28]. Groups were also compared using a SAS^®^ macro implementation of a multiple comparison test based on the method by Elliott and Hynan [29], where, again, a *p*-value of less than 0.05 signified significance. Additionally, orthogonal comparisons were performed by comparing the groups that received varying amounts of CU33FP with the control group. The linear, quadratic, and cubic responses to increasing concentrations of CU33FP were assessed using orthogonal polynomial comparisons [30].

## 3. Results

### 3.1. The Physiochemical Characterizations of CU33FP

Table 2 showed the physicochemical characterizations and nutrient composition of CU33FP. The moisture after fermentation was linearly decreased as the fermentation time increased (*p* < 0.05). The pH reached its highest value at 24 and 48 h fermentation, and *Bacillus*-like bacteria counts were the highest at 24 h fermentation (*p* < 0.05), then reduced at 48 h fermentation and after drying (*p* < 0.05). The neutral protease activity, alkaline protease activity, surfactin, γ-PGA, viscosity, and odor of CU33FP linearly increased as fermentation time increased (*p* < 0.05). The neutral protease activity, γ-PGA, viscosity, and surfactin all increased after drying, whereas the moisture, pH value, bacterial count, and odor decreased (*p* < 0.05).

### 3.2. Growth Performance

Table 3 shows the effects of dietary addition of CU33FP on the growth performance of goat kids. During the preweaning period (0–4 weeks), only the 0.1% CU33FP group showed greater WG than the control group (*p* < 0.05), and the addition of 0.1–0.5% CU33FP could improve FCR compared with the control group (*p* < 0.05). During the postweaning period (4–8 weeks) and 0–8 weeks, the WG and FCR in the groups of added CU33FP were better than the control group. In addition, the results showed linear and quadratic effects as the dietary level of CU33FP increased from 0 to 0.5% (*p* < 0.05).

### 3.3. Health Performance

Table 4 shows the effects of dietary addition of CU33FP on the GHS and fecal bacteria of goat kids. The FCI at 0–4 weeks and 4–8 weeks as well as the fecal bacteria of coliforms at the fourth week were linearly decreased as the dietary level of CU33FP increased (*p* < 0.05). The fecal bacteria of *Bacillus*-likes showed linear and quadratic effects as the dietary level of CU33FP increased at the fourth and eighth weeks (*p* < 0.05). The general health score had a tendency to improve during the postweaning period (4–8 weeks) and 0–8 weeks (*p* < 0.10).

### 3.4. Hematological Traits

Table 5 shows the effects of dietary addition of CU33FP on the blood prolife of goat kids. There was no significant difference among the groups (*p* > 0.05).

### 3.5. Clinical Blood Biochemistry

Table 6 shows the effects of dietary addition of CU33FP on the blood biochemical parameters of goat kids. Serum P, TP, and BUN linearly increased as the dietary level of CU33FP increased (*p* < 0.05), and the 0.3% group had a higher TP compared with that of the control group. In addition, P and BUN in the 0.5% group were significantly higher than those in the 0% group (*p* < 0.05).

### 3.6. Immune Traits

Table 7 shows the effects of dietary addition of CU33FP on the immune traits of goat kids. The oxidative burst of lymphocytes showed a positive linear relationship as the dietary level of CU33FP increased. However, the skin sensitization test showed a quadratic curve, and the group with the addition of 0.1% CU33FP had the lowest performance (*p* < 0.05).

## 4. Discussion

In this experiment, CU33 was identified as *Bacillus amyloliquefaciens*, which is also a kind of probiotic [31]. The European Food Safety Authority (EFSA) has granted the European Union Qualified Presumption of Safety (QPS) qualification for use in feed [32]. It has the ability to produce a variety of extracellular enzymes, such as phytase, α-amylase, cellulase, metalloprotease, and protease [33,34], and also can produce γ-PGA and surfactin [10]. CU33 can secrete neutral and alkaline protease, which increased the pH value by decomposing protein to produce NH_3_, but the pH value decreased after drying (*p* < 0.05). The counts of *Bacillus*-like bacteria reached the highest at 24 h and then decreased after 48 h (*p* < 0.05). Due to the heat production during aerobic fermentation, the moisture gradually decreased from 70% during fermentation. Moisture is an essential medium for microbial fermentation. The reduction of moisture will affect microbe growth. Therefore, the counts of *Bacillus*-like bacteria significantly reduced at 48 h (*p* < 0.05). Fermentation produces γ-PGA, which increases the viscosity, decreases interspace, and reduces oxygen and water activity. Thus, it causes the reduction of the count of *Bacillus*-like bacteria. The number of bacteria and the pH value will reduce after drying. However, the bacteria will produce endospores, and the count of *Bacillus*-like bacteria still has 8.52 log CFU/g feed. The CU33 strain was added to soybean meal for aerobic solid-state fermentation with 70% moisture.

Surfactins can interfere with biofilm growth and cell-to-cell communication and can cause membrane rupture, cell lysis, and disruption of surface properties affecting microbial adhesion [35], which make them have the properties of antimicrobial and antibiofilm agents to be used as an antibiotic alternative [19,36]. The γ-PGA is a polymer formed by the dehydration condensation of α-amino and α-carboxyl groups of two glutamic acid molecules of *Bacillus* strains, and it can promote calcium absorption, cellular immunity, and cytokine production [37,38]. *Bacillus amyloliquefaciens* can produce γ-PGA [10,20], and higher viscosity of the fermented product generally indicates a greater γ-PGA content [39,40], which has been associated with improved calcium absorption and enhanced immune function in animals [37,38]. Compared with the results of fermented feather meal–soybean meal with CU33 [10], it could increase the count of *Bacillus*-like bacteria by 8%, surfactin by 3.4 times, protease activity by 34%, γ-PGA by 2.65 times, and viscosity by 13%. The main reason is that the moisture was increased from 60% to 70%, and the soybean meal contains more soybean hulls, which has better friability and a higher proportion of carbon source, so it is more suitable for *Bacillus amyloliquefaciens* to ferment under aerobic conditions. Xie et al. compared different culture mediums for *Bacillus amyloliquefaciens* 35M fermentation; soybean meal served as a culture medium had a stable and easy-decomposition nitrogen source for the strain to produce enzymes such as protease and amylase [41]. In addition, the production of surfactin required more carbon sources [42]. Compared with the odor of the feather meal–soybean meal served as substrates (3.44) [10], the odor of CU33FP with soybean meal served as the substrates (2.4) was obviously reduced. The main reason is that the CP of soybean meal is lower than that of feather meal, and the fiber content is relatively enhanced, thus reducing its odor.

Adding 5% mixed *Bacillus* spp. fermented feather meal–soybean meal product to the starter could improve the growth performances of goat kids [7]. The authors used CU33 aerobically fermented feather meal–soybean meal for 2 days, and adding 1 or 2% fermented product to the diet could improve the growth performances of goat kids [18]. In this trial, adding 0.1–0.5% CU33FP improved FCR compared with that in the control group, and the 0.1% group had improved WG compared with the control group at 0–4 weeks (*p* < 0.05). During the postweaning period (4–8 weeks) and at 0–8 weeks, the CU33FP addition of 0.1–0.5% improved WG and FCR compared with those in the control group. The main reason is that this fermentation only applied soybean meal as a substrate, further increasing the moisture to 70%, which was suitable for the fermentation of the strain CU33. Therefore, *Bacillus*-like bacteria counts and the content of γ-PGA, surfactin, neutral protease, and alkaline protease were increased. Reducing the CU33FP addition in the starter can improve the growth performance. Adding *Bacillus subtilis* to the diet or milk replacer was beneficial to the growth and immunity of calves and lambs [11,12,13]. Adding *Bacillus amyloliquefaciens*-9 to milk replacer could maintain intestinal microbial homeostasis of Saanen goat kids to improve gut health [14]. Diet supplemented with *Bacillus* spp. fermented feather meal–soybean meal or soybean meal had the effect of improving growth performance in goat kids, growing pigs, and broilers [7,16,17], and the fermented product in this experiment also had the effect on improving weight gain and the feed conversion ratio.

The intake of solid feed before weaning can assist the growth of rumen morphology [5,43] and promote growth performance [7,44]. There are some recommendations about weaning, such as the body weight of the goat kid (e.g., 14–15 kg, or 2–2.5 times of the birth weight), age (at 6–8 weeks of age), or solid feed intake (e.g., starter intake 115–200 g/d, or solid feed intake including hay 30–500 g/d) [1]. However, due to the cost considerations, the goat kids had weaning carried out between 6–12 weeks of age in Taiwan. At the fourth week of this experiment (6 weeks old), the goat kids weighed only 10.5–11.5 kg. The milk replacer was fasted at the fourth week of the experiment (6 weeks old). During the postweaning period (4–8 weeks), the growth performance was affected by CU33FP, and adding 0.1–0.5% had the positive effect.

The lower GHS of young animals is caused by diarrhea, respiratory diseases, and other infections, which lead to higher mortality in feeding [25]. The control group in this trial had a higher number of days of diarrhea. Due to large individual differences within the group, the group of added CU33FP had a tendency to increase compared with the control group. The addition of 0.5% CU33FP can improve FCI and reduce the fecal bacteria of coliforms in preweaning, and increase the fecal bacteria of *Bacillus*-likes around weaning (*p* < 0.05). Coliforms will cause bacterial diarrhea in goat kids, and *Bacillus*-like bacteria counts and surfactin can inhibit coliforms [8,45]. Increasing the addition of CU33FP to the diet enhanced the functional components, such as *Bacillus*-like bacteria counts, protease, γ-PGA, and surfactin, so it had the effect of promoting the health of goat kids to improve the growth performance in preweaning (Table 3).

During the preruminant phase (0–3 weeks of age) is the process of rumen growth, which must rely on feeding dairy products, bypassing the rumen by the esophageal groove. During the transition phase (3–8 weeks of age) and the ruminant phase (above 8 weeks of age), the goat kid can be completely fed solid feed [46]. The weaning time in this trial was at 6 weeks of age, which showed that goat kids can carry out weaning at 6 weeks of age, and the CU33FP groups still had greater WG and FCR. The starter was provided at above 8 weeks of age, and the intake of milk replacer was supplied from 122 g/day/kid at the first week of the experiment to 27.2 g/day/kid at the end week of the experiment. Therefore, the starter intake was increased to stimulate rumen growth, which was beneficial to digestion and absorption.

The values of RBC, WBC, Hgb, Hct, MCV, MCH, MCHC, PLT, Net-s, Lym-L, Mono, Eos, and Baso in the blood were in the normal range of goats [47]. This means that the general health performance, fecal consistency index, and *Bacillus*-like bacteria counts of the control group were all within the controllable healthy range. Due to large individual differences among goat kids, it caused no significant difference on blood profiles among the groups *(p* > 0.05). BUN is a metabolite of protein in animals, and its content is affected by not only the synthesis and excretion of animals, but also factors such as species, diet or disease. In this experiment, the dietary protein of each group was equal, and BUN of goat kids was linearly increased as the dietary level of CU33FP increased. The reason is that the macromolecule protein in the substrate transformed into small molecule peptides or amino acids after CU33FP fermented by microbes, which make the digestion and absorption of proteins or amino acids better. TP in the blood was linearly enhanced as the dietary level of CU33FP increased. When animals are in the rapid growth stage, the decomposition of osteoclast in the bones enhances the ALP activity and P content in the blood [47]. This is consistent with the results of this experiment that fed goat kids 0.5% CU33FP had a higher ALP activity (*p* < 0.10) and P content (*p* < 0.05) in the blood. This corresponded with the result of the 0.5% CU33FP group improving the WG and FCR compared with those of the control group during the postweaning period (4–8 weeks) and at 0–8 weeks. It proved that CU33FP has the effect of improving protein digestion and utilization to promote growth in goat kids.

*Bacillus amyloliquefaciens* is a probiotic with the potential to be a feed additive. In the animal experiments of goat kids, it has the effect of regulating the number of lymphocytes, the oxidative burst activity of lymphocytes, the phagocytosis activity of lymphocytes, and the concentration of immunoglobulin, thereby enhancing the infection resistance and improving the growth [42,48,49,50]. The ROS produced by the oxygen burst of lymphocytes can destroy the phagocytized pathogens, which is an important innate immune response, and this ability will affect the subsequent innate and adaptive immune responses [51,52,53]. The skin sensitization test of PHA-P is used to evaluate cellular immunity, and the increasing values are associated with the increased leukocyte activity [54,55]. This study found that the goat kids’ diet supplemented with CU33FP can enhance the average fluorescence intensity of lymphocyte oxygen burst and the skin sensitization test performance. It was confirmed that CU33FP can improve the innate immune responses such as lymphocyte oxygen burst and leukocyte activity. This may be one of the reasons for the improvement of growth performance and diarrhea index in this study.

## 5. Conclusions

Adding 0.1% CU33 fermented soybean meal product into the diet can increase the count of Bacillus-like fecal bacteria while reducing coliform bacteria in goat kids. Additionally, this product enhances the oxygen burst of macrophages, aiding in the regulation of immunity. As a result, it helps improve diarrhea in goat kids when weaning and promotes their growth.

## Figures and Tables

**Table 1:** The composition of starter.

Ingredients, %	0%	0.1%	0.3%	0.5%
Alfafa meal	15	15	15	15
Yellow corn, grain	55.1	55.1	55.1	55.1
Soybean meal, CP 43%	21.5	21.4	21.2	21.0
Fish meal, CP 65%	2.0	2.0	2.0	2.0
CU33FP, CP 44%	0	0.1	0.3	0.5
Sugar cane molasses	3.0	3.0	3.0	3.0
Dicalcium phosphate	1.2	1.2	1.2	1.2
Limestone, pulverized	1.2	1.2	1.2	1.2
Salts	0.7	0.7	0.7	0.7
Vitamin Premix ^1^	0.2	0.2	0.2	0.2
Mineral Premix ^2^	0.1	0.1	0.1	0.1
Total	100	100	100	100
Calculated value				
CP, %	17.14	17.14	17.14	17.14
ME, Mcal/kg	2932	2932	2932	2932
Ca, %	1.07	1.07	1.07	1.07
P, %	0.61	0.61	0.61	0.61
Analyzed value, %				
Crude protein	17.70	17.81	17.90	17.89
Calcium	1.04	1.07	1.06	1.05
Total phosphorus	0.66	0.65	0.62	0.64

^1^ Vitamin premix supplied per kilogram of diet: vitamin A, 1,000,000 IU; vitamin D_3_, 270,000 IU; vitamin E, 2900 IU. ^2^ Mineral premix supplied per kilogram of diet: Cu, 5000 mg; Fe, 9000 mg, Zn, 8000 mg, Mn, 6000 mg; Co (CoCO_3_, 49.5% Co) and Se (Na_2_SeO_3_, 45.7% Se), 0.25 mg.

**Table 2:** Physical and chemical analysis of CU33FP ^1^.

Items	Fermentation Time				SEM	*p*-Value
	0 h	24 h	48 h	Dry		
Moisture	70.4 ^a^	60.8 ^b^	50.5 ^c^	10.3 ^d^	0.29	<0.001
pH	6.39 ^b^	7.51 ^a^	7.71 ^a^	6.58 ^b^	0.10	<0.001
*Bacillus*-like, log CFU/g feed	7.40 ^d^	9.22 ^a^	9.03 ^b^	8.52 ^c^	0.04	<0.001
Neutral protease activity, U/g	25.3 ^d^	377 ^c^	542 ^b^	670 ^a^	5.4	<0.001
Alkaline protease activity, U/g	32.7 ^c^	361 ^b^	568 ^a^	566 ^a^	5.4	<0.001
Surfactin, mg/g	-	3.71 ^c^	4.25 ^b^	5.49 ^a^	0.05	<0.001
γ-PGA, %	-	2.13 ^c^	3.81 ^b^	5.33 ^a^	0.03	<0.001
Viscosity, score	-	1.64 ^c^	2.13 ^b^	2.65 ^a^	-	<0.001
Odor, score	-	3.11 ^b^	3.42 ^a^	2.40 ^c^	-	<0.001

^1^ The data are given as mean, n = 3. ^a–d^ Means in the same row with different superscripts differ significantly (*p* < 0.05).

**Table 3:** Effects of dietary addition of CU33FP on growth performances of goat kids.

Items	0%	0.1%	0.3%	0.5%	SEM	*p*-Value	Orthogonal Contrast ^1^
Body weight, kg							
Initial, 0 week	5.86	5.65	5.73	5.69	0.12	0.63	-
Weaning, 4 week	10.5	11.5	11.1	11.3	0.30	0.11	-
Final, 8 week	15.2 ^b^	17.8 ^a^	17.7 ^a^	17.5 ^a^	0.31	<0.01	L, Q
Preweaning period (0–4 weeks)							
Total feed intake, kg/kid	11.5	12.2	11.5	11.7	0.35	0.39	-
Starter intake, kg/kid	9.92	10.6	9.85	10.06	0.35	0.37	-
Weight gain, kg/kid	4.64 ^b^	5.85 ^a^	5.37 ^ab^	5.61 ^ab^	0.25	0.01	L
Feed conversion rate (feed intake/weight gain)	2.47 ^a^	2.08 ^b^	2.14 ^b^	2.09 ^b^	0.08	0.04	L, Q
Postweaning period (4–8 weeks)							
Starter intake, kg, kg/kid	18.4	19.4	18.8	18.6	0.45	0.43	-
Weight gain, kg/kid	4.71 ^b^	6.30 ^a^	6.61^a^	6.20 ^a^	0.23	0.001	L, Q
Feed conversion rate (starter intake/weight gain)	3.91 ^a^	3.08 ^b^	2.84 ^b^	3.02 ^b^	0.14	0.001	L, Q
0–8 weeks experimental period							
Total feed intake, kg/kid	29.9	31.6	30.3	30.2	0.65	0.26	-
Starter intake, kg/kid	28.3	30.0	28.7	28.6	0.65	0.26	-
Weight gain, kg/kid	9.34 ^b^	12.2 ^a^	12.0 ^a^	11.8 ^a^	0.31	0.001	L, Q
Feed conversion rate (feed intake/weight gain)	3.20 ^a^	2.60 ^b^	2.53 ^b^	2.56 ^b^	0.07	0.001	L, Q

^a,b^ Means in the same row with different superscripts differ significantly (*p* < 0.05). Total feed intake = milk replacer + starter. ^1^ L indicates that the linear effect of treatment is significant (*p* < 0.05); Q indicates that the quadratic effect of treatment is significant *(p* < 0.05); - means the values in the same row are not significantly different (*p* > 0.05).

**Table 4:** Effects of dietary addition of CU33FPon GHS and fecal bacteria of goat kids.

Period	0%	0.1%	0.3%	0.5%	SEM	*p*-Value	Orthogonal Contrast ^1^
	General health score (n = 10)						
Preweaning period (0–4 weeks)	17.7	23.5	23.8	22.5	2.71	0.36	-
Postweaning period (4–8 weeks)	19.5	25	26.3	25.3	1.99	0.085	-
Experimental period (0–8 weeks)	37.2	48.5	50.1	47.8	3.79	0.083	-
	Fecal consistency index, % (n = 10)						
Preweaning period (0–4 weeks)	39.5 ^a^	35.0 ^ab^	32.8 ^ab^	32.2 ^b^	1.90	0.04	L
Postweaning period (4–8 weeks)	35.4 ^a^	30.1 ^ab^	29.4 ^ab^	28.0 ^b^	1.70	0.02	L
	Fecal bacteria *, log CFM/g						
Exp. 4 weeks							
Coliforms	5.94 ^a^	3.86 ^b^	4.17 ^ab^	3.84 ^b^	0.45	0.011	L
*Bacillus*-likes	3.13 ^b^	3.82 ^ab^	4.71 ^a^	4.77 ^a^	0.34	0.007	L
Exp. 8 weeks							
Coliforms	4.14	4.83	4.67	3.96	0.39	0.21	-
*Bacillus*-likes	1.2 ^b^	2.49 ^a^	2.51 ^a^	2.41 ^a^	0.26	0.004	L, Q

^a,b^ Means in the same row with different superscripts differ significantly (*p* < 0.05). ^1^ L indicates that the linear effect of treatment is significant (*p* < 0.05); Q indicates that the quadratic effect of treatment is significant (*p* < 0.05); - means the values in the same row are not significantly different (*p* > 0.05). * n = 6.

**Table 5:** Effects of dietary addition of CU33FP on blood prolife of goat kids.

Items	0%	0.1%	0.3%	0.5%	SEM
WBC, /uL	11,545	11,531	11,533	10,417	869
RBC, M/uL	3.32	3.53	3.25	3.26	0.12
Hgb, gm/L	11.2	11.7	10.8	10.5	0.4
Hct, %	35.7	35.3	33.9	32.9	1.8
MCV, fL	103.6	97.6	104.6	101.4	3.5
MCH, pg	32.7	32.0	33.4	32.1	1.0
MCHC, %	31.7	32.4	32.1	31.4	1.4
PLT, ×10^3^/uL	729	747	713	713	28
WBC classification					
Net-s, %	17.9	21.5	20.9	20.7	1.4
Lym-L, %	75.1	71.8	71.5	72.6	1.6
Mono, %	5.04	5.11	5.85	5.36	0.41
Eos, %	1.38	1.44	1.24	0.94	0.17
Baso, %	0.44	0.44	0.50	0.41	0.10

n = 10. WBC = white blood cell; RBC = red blood cell; Hgb = hemoglobin; Hct = hematocrite; MCV = mean corpuscular volume; MCH = mean corpuscular hemoglobin; MCHC = mean corpuscular hemoglobin concentration; PLT = platelet; leukocyte; Net-s = neutrophil extracellular traps; Lym-L = lymphocytes; Mono = monocytes; Eos = eosinophils; Baso = basophils.

**Table 6:** Effects of dietary addition of CU33FP on clinical blood biochemistry of goat kids.

Items	0%	0.1%	0.3%	0.5%	SEM	*p*-Value	Orthogonal Contrast ^1^
AST, U/L	396	435	409	337	29.2	0.13	-
ALT, U/L	43.4	34.5	34.0	37.5	2.84	0.09	-
γ-GT, U/L	25.5	28.4	27.0	29.4	1.9	0.5	-
LDH, U/L	3205	3090	2552	2982	194	0.12	-
CK, U/L	10,960	9036	11,777	10,876	1283	0.5	-
ALP, U/L	1426	2034	1841	2588	317	0.1	-
ACP, U/L	0.29	0.14	0.24	0.25	0.08	0.61	-
Ca, mg/dL	9.87	9.63	10.30	9.76	0.42	0.71	-
P, mg/dL	7.73 ^b^	7.91 ^ab^	8.29 ^ab^	8.80 ^a^	0.24	0.02	L
Glucose, mg/dL	63.1	77.6	76.5	71.1	4.1	0.07	-
Cholesterol, mg/dL	74.3	66.4	64.6	64.1	3.4	0.15	-
TG, mg/dL	24.4	23.6	22.0	21.1	1.5	0.43	-
Total protein, g/dL	6.71 ^b^	6.98 ^ab^	7.74 ^a^	7.85 ^a^	0.24	0.004	L
ALB, g/dL	2.86	2.41	2.90	2.90	0.28	0.52	-
GLO, g/dL	3.85	4.56	4.84	4.95	0.38	0.19	-
A/G	0.84	0.58	0.69	0.65	0.14	0.6	-
BUN, mg/dL	13.5 ^b^	17.4 ^ab^	17.1 ^ab^	21.1 ^a^	1.2	0.002	L
Creatinine, mg/dL	1.00	1.01	1.01	1.13	0.08	0.74	-

n = 10. ^1^ L indicates that the linear effect of treatment is significant (*p* < 0.05); Q indicates that the quadratic effect of treatment is significant (*p* < 0.05); -^a,b^ Means in the same row with different superscripts differ significantly (*p* < 0.05).

**Table 7:** Effects of dietary addition of CU33FP on immune traits of goat kids.

Items	0%	0.1%	0.3%	0.5%	SEM	*p*-Value	Orthogonal Contrast ^1^
4 weeks							
Mean fluorescence intensity							
Phagocytosis	1.87	2.52	2.30	3.05	0.37	0.19	-
Oxygen burst	888 ^b^	867 ^b^	1091 ^ab^	1158 ^a^	67	0.01	L
Lymphoblastogenesis, specific fluorescence							
PHA	14,015	11,975	9714	9399	1326	0.07	-
LPS	8183	7276	5686	5028	978	0.12	-
PMA/ION ^2^	8742	7532	6309	5100	1037	0.11	-
Swelling degree, mm							
Swelling	0.61 ^b^	3.26 ^a^	2.55 ^ab^	1.88 ^ab^	0.53	0.01	Q
Immunoglobulin, mg/dL							
IgA	0.69	0.71	0.56	0.66	0.05	0.16	-
IgG	4.18	4.23	3.89	4.63	0.22	0.88	-
IgM	0.43	0.44	0.40	0.43	0.06	0.15	-
8 weeks							
IgA	0.70	0.67	0.66	0.70	0.04	0.64	-
IgG	2.79	2.36	2.13	2.71	0.32	0.95	-
IgM	0.21	0.36	0.28	0.22	0.05	0.16	-

n = 10. ^a,b^ Means in the same row with different superscripts differ significantly (*p* < 0.05). ^1^ L indicates that the linear effect of treatment is significant (*p* < 0.05); Q indicates that the quadratic effect of treatment is significant (*p* < 0.05); ^2^ PMA/ION: Phorbol-12-myristate-13-acetate/Ionomycin. - means the values in the same row are not significantly different (*p* > 0.05).

## Data Availability

The data presented in this study are available upon request from the corresponding author.
